# Imaging grain boundaries in a two-dimensional polymer

**DOI:** 10.1038/s42004-020-00380-3

**Published:** 2020-10-07

**Authors:** Victoria Richards

**Affiliations:** Communications Chemistry, https://www.nature.com/commschem/

## Abstract

Organic materials are highly sensitive to electron beam irradiation and thus easily damaged upon imaging by electron microscopy. Now, low-dose aberration-corrected high resolution transmission electron microscopy allows for less invasive near-atomic-scale imaging of a two-dimensional polymer.

Synthetic two-dimensional polymers have shown promise for a variety of applications. However, little is understood about the grain boundaries present within these materials, which may play important roles in dictating their properties. Now, an international team led by Ute Kaiser and Haoyuan Qi from Universität Ulm, and Xinliang Feng and Thomas Heine from Technische Universität Dresden achieve near-atomic-scale visualization of the grain boundaries in a layer-stacked 2D polyimine using aberration-corrected high-resolution transmission electron microscopy (AC-HRTEM) (10.1126/sciadv.abb5976)^1^.

Two-dimensional materials are prone to structural disintegration upon irradiation with an electron beam. Their high-resolution imaging has thus proven to be a challenging goal for electron microscopists over recent years. “From our experience with inorganic 2D materials, we have gained valuable insights into damage mechanisms, and developed methodologies to significantly reduce electron radiation damage”, says Kaiser. “However, compared to beam-sensitive inorganic materials such as graphene and transition metal dichalcogenides, the susceptibility of 2D polymers towards electron radiation is several orders of magnitude higher. Therefore, TEM imaging of 2D polymers—even just seeing the lattice fringes—seemed to be a formidable task”, she explains.

Nonetheless, by balancing the electron dose and the image contrast, the team successfully determined an experimental procedure for AC-HRTEM that allows for direct grain boundary observation in a 2D polymer with a resolution of 2.3 Å. Porphyrin nodes can be distinguished from terephthalaldehyde linkers in both the pristine 2D network and at defect sites. The researchers identified a number of distinct grain boundary types, including antiphase boundaries as well as low-angle and high-angle grain boundaries (Fig. 1). Antiphase boundaries are proposed to result from self-corrections applied to missing-linker and missing-node errors that occur during polymerization, while low-angle and high-angle grain boundaries are attributed to the coalescence of stochastically oriented grains during crystal growth.

**Fig. 1:**
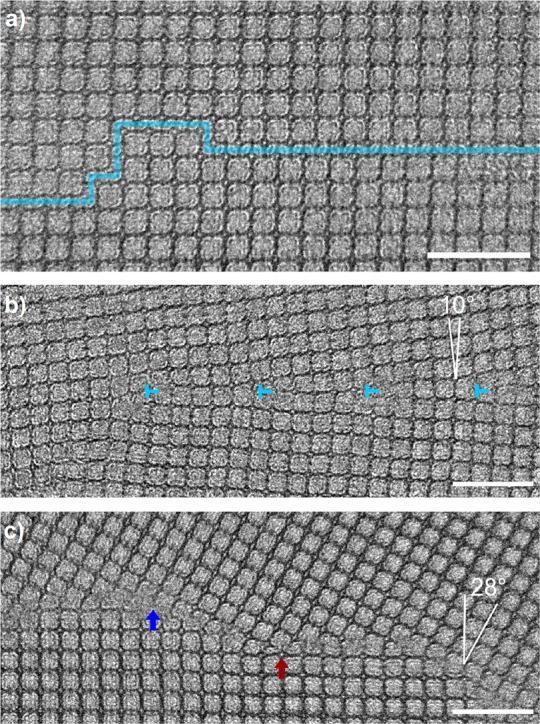
Grain boundaries in a 2D polyimine identified from aberration-corrected high-resolution transmission electron microscopy. **a** An antiphase boundary; the blue line follows the boundary. **b** A low-angle grain boundary with a misorientation of 10 degrees; dislocations are marked in blue. **c** A high-angle grain boundary with a misorientation of 28 degrees; the red arrow marks a porphyrin termination and the blue arrow marks a terephthalaldehyde termination. Scale bars: 10 nm. Adapted from Sci. Adv., **6**, 33, eabb5976 (2020) DOI: 10.1126/sciadv.abb5976.

“Without direct observation, one could speculate that all grain boundaries, regardless of whether in organic or inorganic materials, should always look the same. But based on our observations, we can say with certainty that they are not”, comments Kaiser. While inorganic 2D materials display high-energy point defects at their grain boundaries, both experiment and theory demonstrate that the crystalline domains of these 2D polymers tend to be connected via flexible covalent bonds. This may suggest a higher degree of local robustness in comparison to inorganic materials.

‘All these details could offer a realistic starting point for further theoretical modelling and experimental endeavors, such that the structure—property correlation in 2D polymers can be better understood’, concludes Kaiser. The team’s next goal is to achieve atomic resolution, which they hope would allow them to identify different functional groups and to observe the chemical environment in the network’s pores.
